# Papillary thyroid carcinoma presenting as paraneoplastic acral vascular syndrome in a 37-year-old female: A case report

**DOI:** 10.5339/qmj.2026.44

**Published:** 2026-06-16

**Authors:** Muhammad Bilal Jamshaid, Afia Aziz, Huma Munir, Sarah Obiedat, Samir Alhyassat, Adel AlBozom, Basem Awadh, Samar AlEmadi

**Affiliations:** 1 Department of Rheumatology, Hamad Medical Corporation, Doha, Qatar; 2 Department of Internal Medicine, Phoenixville Hospital, PA, USA; 3 Department of Internal Medicine, Hamad Medical Corporation, Doha, Qatar; 4 Department of Internal Medicine, Sheikh Zayed Hospital, Lahore, Pakistan; 5 Department of Pathology, Hamad Medical Corporation, Doha, Qatar

**Keywords:** Digital ischemia associated with cancer (DIAC), paraneoplastic acral vascular syndrome, Raynaud’s phenomenon, peripheral gangrene, thyroid malignancy, papillary thyroid cancer

## Abstract

**Background:**

Paraneoplastic acral vascular syndrome is a rare condition associated with various malignancies, most commonly adenocarcinomas. It manifests as digital ischemia, including Raynaud’s phenomenon, acrocyanosis, and digital gangrene. Reported cases typically show improvement after treatment of the underlying cancer. We describe the first known case of paraneoplastic acral vascular syndrome secondary to papillary thyroid carcinoma (PTC), with complete resolution following thyroidectomy.

**Case presentation:**

A 37-year-old previously healthy woman was referred from the vascular surgery clinic for evaluation of episodic digital discoloration. She reported a four-year history of recurrent Raynaud-like color changes affecting the middle fingers of both hands and the lateral four toes of both feet. She denied symptoms suggestive of connective tissue disease, inflammatory arthritis, autoimmune disorders, or cardiovascular disease, as well as tobacco or alcohol use. On examination, her feet were cold, with ulcers on the first and second toes of the right foot. Peripheral pulses in the lower extremities were diminished, with bilaterally weak dorsalis pedis pulses, more pronounced on the right.

Her autoimmune panel was positive for anti-nuclear, anti-dsDNA, and anti-centromere antibodies. Capillaroscopy and arterial Doppler ultrasound of the lower limbs were unremarkable. Her symptoms progressed despite multiple treatments, leading to gangrene of the first and second toes of the right foot. A positron emission tomography (PET) scan showed no evidence of vasculitis but revealed focal uptake in a 1.14 cm nodule in the right thyroid lobe. Fine-needle aspiration suggested PTC. She underwent thyroidectomy, and histopathology confirmed papillary carcinoma along with medium-sized vessels showing intimal thickening and marked luminal narrowing, consistent with vasculitis. At 18month followup, she demonstrated complete clinical resolution with no new ulcerations.

**Conclusion:**

Paraneoplastic acral vascular syndrome is a rare condition that resembles Raynaud’s phenomenon and may improve after treatment of the associated cancer. This case emphasizes the importance of considering occult cancer in patients with unexplained digital ischemia.

## 1. INTRODUCTION

Raynaud’s syndrome is a relatively common condition that may occur as a primary disorder or secondary to other diseases, including autoimmune conditions such as scleroderma, systemic lupus erythematosus, and antiphospholipid syndrome, as well as hyperviscosity syndromes and certain medications. However, there are other conditions that mimic Raynaud’s and should be differentiated from it for effective patient management and prevention of complications such as digital ischemia and amputation. One such condition is paraneoplastic acral vascular ischemia (PAVS).

It is estimated that the incidence of PAVS is 2 cases per 100,000 patients per year. The median time from the onset of PAVS to tumor diagnosis is 2.5 months. It typically manifests as painful digital ischemia, mainly affecting the fingers or toes, with presentations ranging from acrocyanosis and Raynaud’s phenomenon to gangrene. Unusual features suggestive of malignancy mainly include sudden onset in patients above 50 years of age, associated with weight loss, and the exclusion of other related vascular causes.^1–3^

The exact pathophysiology remains unclear; however, proposed mechanisms include a vaso-occlusive process with sympathetic hyperactivity—either due to mechanical invasion of sympathetic nerves by the tumor, the production of vasoconstrictive substances by tumor cells, an immune complex-mediated reaction, or impaired hemostasis.^4^ The term “paraneoplastic acral vascular ischemia” was first coined by Poszepczynska-Guigné and colleagues in 2002. Prior to this, the condition had been described as paraneoplastic Raynaud’s phenomenon or digital ischemia associated with malignancy. The earliest description dates back to O’Conner in 1884. Since then, the syndrome has been linked to a variety of cancers, with adenocarcinomas being the most frequently reported—accounting for approximately 41–46% of cases and commonly involving the lungs and ovaries. Other associated malignancies include hematological cancers, such as lymphomas and leukemias, as well as anaplastic tumors.^2,5–7^

Our literature review indicates that this is the first documented case of paraneoplastic acral vascular syndrome associated with papillary thyroid carcinoma (PTC).^1–7^

## 2. CASE PRESENTATION

A 37-year-old previously healthy woman was referred from the vascular surgery clinic in July 2022 for evaluation of episodic discoloration of the fingers and toes. She reported recurrent color changes affecting the middle fingers of both hands and the lateral four toes of both feet. These episodes were triggered by cold exposure, during which the digits turned white and blue, and improved with rewarming. These symptoms had been ongoing for four years. She also reported right leg and foot pain after walking less than 10 m. In addition, she had developed wounds on the first and second toes of the right foot over the preceding two months, following minor trauma. She denied joint pain, skin rash, skin tightening, sicca symptoms, and gastrointestinal or genitourinary complaints. She also denied tobacco and alcohol use.

On physical examination, a dry necrotic wound measuring 1×1 cm was present on the right first toe, along with a 0.8×0.5 cm necrotic wound on the second toe. The right second toe showed complete discoloration (Figure 1). The right foot was cold to the touch, and dorsalis pedis pulses were weak bilaterally, more diminished on the right than the left (R>L).

Her complete blood count, comprehensive metabolic panel, and inflammatory markers were unremarkable; however, her autoimmune profile was positive for anti-nuclear antibody (ANA) at a titer of 1:1,280 with a centromere pattern (normal <1:40), anti-double-stranded deoxyribonucleic acid (anti-dsDNA) antibody of 19 IU/mL (normal <14 IU/mL), and anti-centromere antibody (CENP) of 240 U/mL (normal <7 U/mL). The remaining extractable nuclear antigen (ENA) panel was negative (Table 1). Capillaroscopy was performed to assess capillary changes associated with Raynaud’s phenomenon, but it did not reveal significant findings indicative of secondary Raynaud’s. An arterial Doppler ultrasound of the limbs was also performed to evaluate arterial narrowing, revealing early and extensive atherosclerotic changes in the blood vessels.

Meanwhile, she was started on aspirin, calcium channel blockers, and a glyceryl trinitrate patch by the vascular surgery team, which did not lead to improvement. She was also followed by podiatry for wound care.

Her magnetic resonance angiography (MRA) of the aorta and lower limbs showed occlusion of the bilateral posterior tibial arteries and poor distal runoff via collateral vessels (Figure 2). A PET scan was performed to check for signs of active vasculitis, but none were found in the lower extremities or elsewhere. However, it showed focal uptake in the right thyroid lobe, suggestive of a possible adenoma. Thyroid ultrasound confirmed this finding, revealing a right thyroid lobe nodule measuring 1.14 cm, along with multiple bilateral cervical lymph nodes.

Thyroid nodule histopathology was obtained via fine-needle aspiration (FNA), which showed findings suggestive of PTC. Meanwhile, the patient’s right foot wound continued to worsen and became gangrenous. She underwent computed tomography (CT)-guided chemical lumbar sympathectomy as advised by the vascular surgeon, but it did not result in clinical improvement.

Eventually, the patient underwent thyroidectomy. Macroscopic examination revealed an infiltrative 1.5 cm nodule in the right lobe with a solid, whitish appearance. Histologically, the nodule exhibited papillary architecture and psammomatous calcifications (Figure 3a). The nuclei were enlarged with clearing, grooves, and pseudo-inclusions, confirming the diagnosis of PTC. Further microscopic examination of the tissue revealed medium-sized blood vessels with intimal thickening and severe luminal narrowing (Figure 3b). Associated calcifications and destruction of the internal elastic lamina were also present (Figures 3c and d). Clusters of macrophages were observed around the area, forming multinucleated giant cells (Figures 3e and f). Histological features raised the possibility of vasculopathy.

After thyroidectomy, the remnant thyroid tissue in the thyroid bed was treated with radioiodine ablation (RAI) using 1,114 MBq (megabecquerels) of I-131. However, during this period, her gangrene worsened, and she received five days of intravenous alprostadil, after which her wound showed partial improvement. Following RAI, she experienced fewer episodes of Raynaud’s phenomenon. She was advised to keep herself warm to avoid further episodes of Raynaud’s and was maintained on aspirin and pentoxifylline. Follow-up after one year of thyroidectomy showed that her wound on the right first toe had healed, with significant improvement in the right second toe and no new ulcerations (Figure 4). At 18-month follow-up, she demonstrated complete clinical resolution, with no new ulcerations.

## 3. DISCUSSION

The clinical picture of this patient suggests paraneoplastic acral vascular syndrome. The underlying pathophysiology of PAVS is not fully understood, but multiple factors, such as a vaso-occlusive process, an immune complex-mediated reaction, and impaired hemostasis, may be involved.^5^ In our patient, the histology of the thyroid tissue showed features of vasculitis, which is consistent with the findings of Ewa et al.’s case series, in which five patients were found to have heterogeneous inflammatory cell infiltrates with intimal proliferation in skin biopsies, favoring an immune-mediated mechanism with vasculitis as a probable cause of inflammation.^7^

Our patient presented with episodic discoloration involving the middle fingers of both hands and the lateral four toes bilaterally—an atypical pattern for primary Raynaud’s phenomenon. Examination revealed weak bilateral dorsalis pedis pulses, and in the absence of any prior history of peripheral vascular disease, subsequent evaluation identified an underlying malignancy. This presentation parallels the case reported by Maharaj et al.^8^ in which Raynaud’s phenomenon was the initial manifestation of pancreatic cancer, as well as previous reports describing Raynaud’s phenomenon with digital ulcers preceding the diagnosis of lung adenocarcinoma.^9,10^

Unlike previous cases with negative autoimmune workups, our patient tested positive for ANA, anti-dsDNA, and anti-centromere antibodies. However, she lacked clinical features of connective tissue disease or inflammatory arthritis, such as photosensitivity, oral ulcers, skin tightening, arthritis, or calcinosis. Moreover, nailfold capillaroscopy findings were normal, effectively ruling out systemic sclerosis and other autoimmune conditions. Distinctively, the thyroid carcinoma in our patient exhibited histopathological evidence of vasculopathy, underscoring a paraneoplastic vascular mechanism.

Given the patient’s age and gender, along with occlusion of the bilateral posterior tibial arteries, Buerger’s disease was initially considered in the differential diagnosis. However, the absence of a history of smoking or passive smoke exposure made this diagnosis unlikely.^10,11^

Paraneoplastic acral vascular syndrome is a rare condition with limited literature, and most available data are based on case reports and case series. It is associated with various malignancies, particularly adenocarcinomas, followed by lymphomas. Adenocarcinomas of the lung, ovary, and stomach account for approximately 60% of all associated neoplasms and are almost always in advanced stages with metastasis. The remaining neoplasms include sarcomas, hematologic neoplasms, and tumors of unknown origin. In about 50% of cases, skin lesions precede the diagnosis of cancer.^3,11^

PAVS is most commonly found in elderly women, based on the current literature, with a median age of 55–56 years at onset. This contrasts with our case, which had a relatively younger age at onset. Moreover, women are more likely to have an associated malignancy than men, with a ratio of 1.14.^3,12^

Furthermore, during our literature review,^1–5,11–13^ we identified that multiple medical and surgical treatments have been attempted for PAVS, including the use of aspirin, anticoagulants, calcium channel blockers, nitrates, prostacyclin, and steroids. Most of the treatments were noted to have little effect, except for prostacyclins and antiplatelet agents, where partial improvement was noted. However, the treatment of the underlying malignancy was associated with remission of digital symptoms in most cases. In this case, the patient’s symptoms improved, with no further ulcerations.^13^

PubMed and Google databases were searched using the keywords paraneoplastic acral vascular syndrome, paraneoplastic acrovascular syndrome, paraneoplastic Raynaud’s phenomenon, and paraneoplastic digital ischemia. No reported cases of paraneoplastic acral vascular syndrome associated with papillary thyroid carcinoma were identified, which makes our case unique. Papillary thyroid carcinoma is rarely associated with paraneoplastic syndromes, with only a handful of documented cases involving polymyositis, lupus, SIADH, and neurological paraneoplastic syndromes.^13–17^ However, it is crucial to remain vigilant and consider these possibilities when evaluating patients with thyroid malignancies.

## 4. CONCLUSION

Paraneoplastic acral vascular syndrome is a rare Raynaud’s mimic that can signal an underlying malignancy. This case demonstrates that progressive digital ischemia may occur even in the absence of autoimmune disease or traditional vascular risk factors. The patient’s complete resolution after thyroidectomy supports a direct paraneoplastic mechanism. By identifying the first association with PTC, this report broadens the spectrum of related cancers and reinforces the importance of considering occult malignancy in cases of unexplained or refractory digital ischemia.

## CONSENT FOR PUBLICATION

Written informed consent was obtained from the patient for publication of this case report.

## ACKNOWLEDGMENT

The authors would like to thank the patient for consenting to the publication of this case report. No other acknowledgments are applicable.

## FUNDING

The author(s) received no financial support for the research, authorship, and/or publication of this article.

## AUTHORS’ CONTRIBUTION

MJ followed and managed the case and wrote the case report. AZ and HM contributed to the case report writing. SO, SA, and AB assisted with the histopathology slides and their interpretation. BA and SA contributed to the management of the case.

## COMPETING INTERESTS

The author(s) declare that there is no conflict of interest.

## DISCLOSURE OF AI USE

ChatGPT (OpenAI) was used solely to assist with proofreading for grammar and spelling.

## Figures and Tables

**Figure 1.**
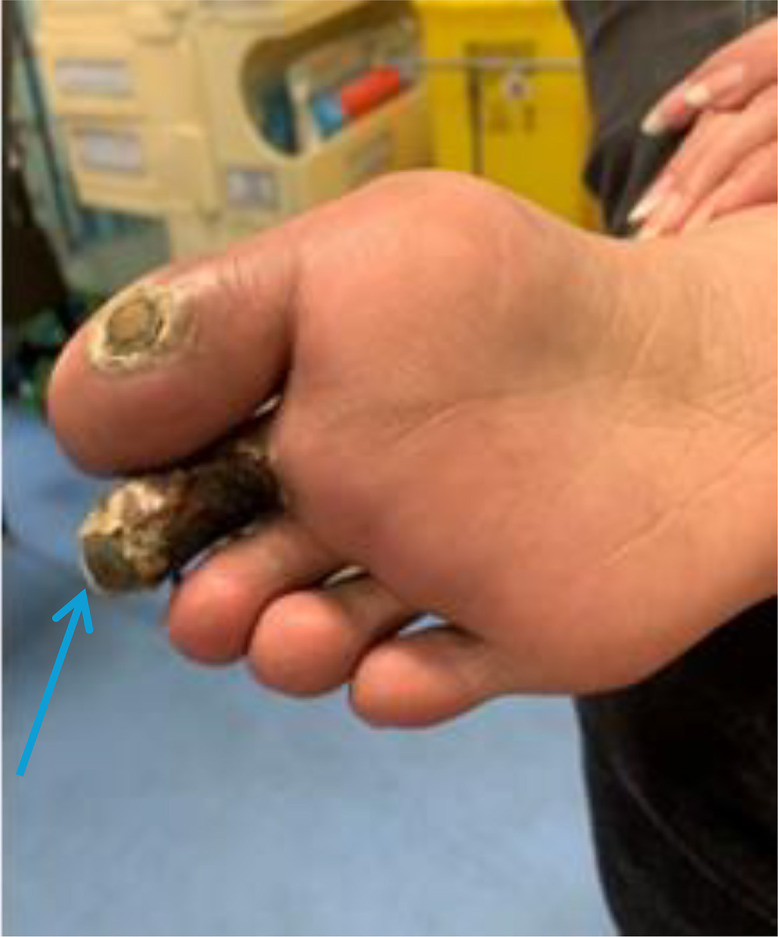
Dry necrotic wound of 1×1 cm (black arrow) on the first toe with well-defined margins. Discoloration of the right second toe with a 0.8×0.5 cm necrotic wound and eschar (blue arrow).

**Figure 2.**
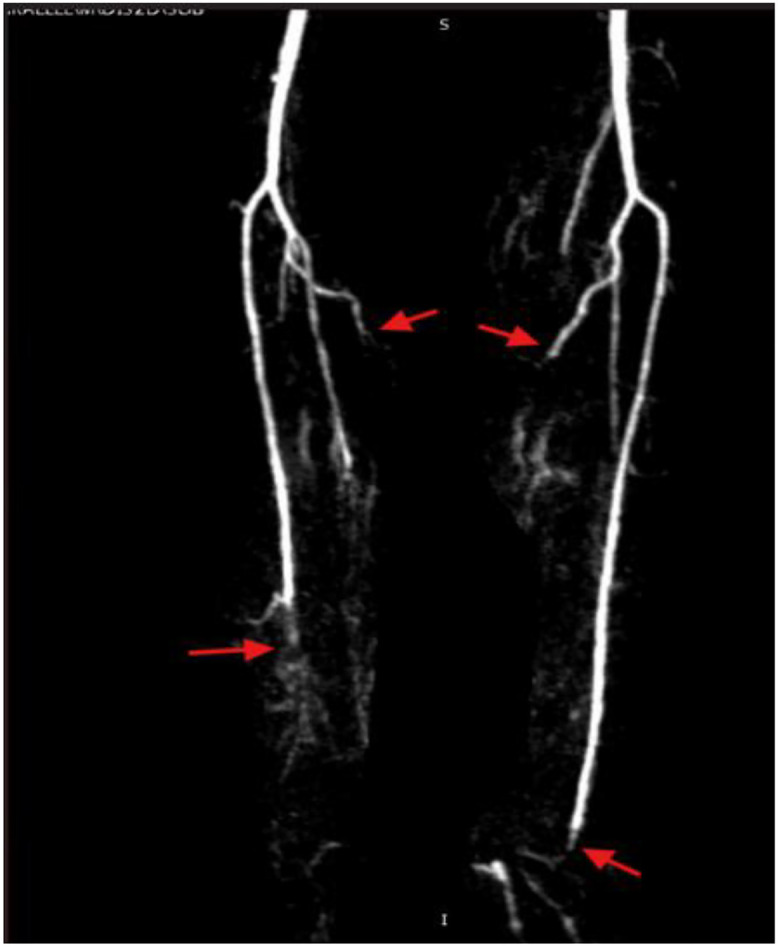
Total occlusion of the bilateral tibial arteries and irregular luminal narrowing of the distal anterior tibial arteries (red arrow).

**Figure 3.**
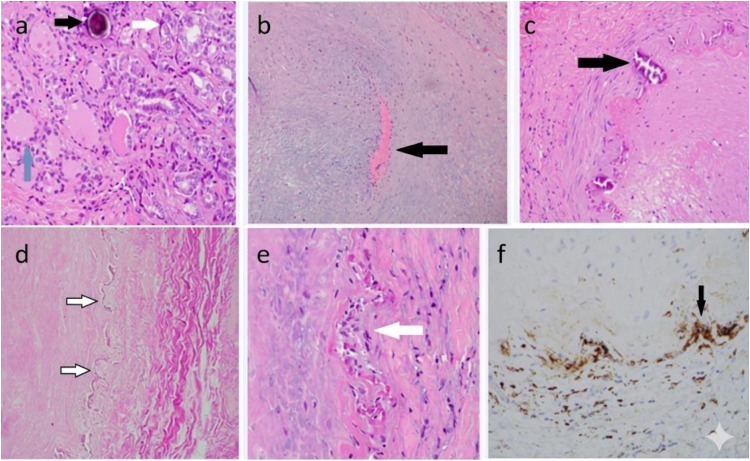
(a) Normal thyroid tissue (gray arrow) with adjacent PTC (white arrow) and associated psammoma bodies (black arrow) (Hematoxylin and Eosin (H&E) stain, 40×). (b) Medium-sized vessel with intimal thickening and severe narrowing of the lumen (black arrow) (H&E, 10×). (c) Vessel wall with calcifications (black arrow) (H&E, 20×). (d) Elastic Van Gieson stain highlighting fragmentation of the internal elastic lamina (white arrow) (Elastic Van Gieson (EVG) stain, 20×). (e) Aggregates of macrophages and a multinucleated giant cell infiltrating the vessel wall (white arrow) (H&E, 40×). (f) Immunohistochemical staining for CD68 highlights macrophages within the vessel wall (black arrow) (40×).

**Figure 4.**
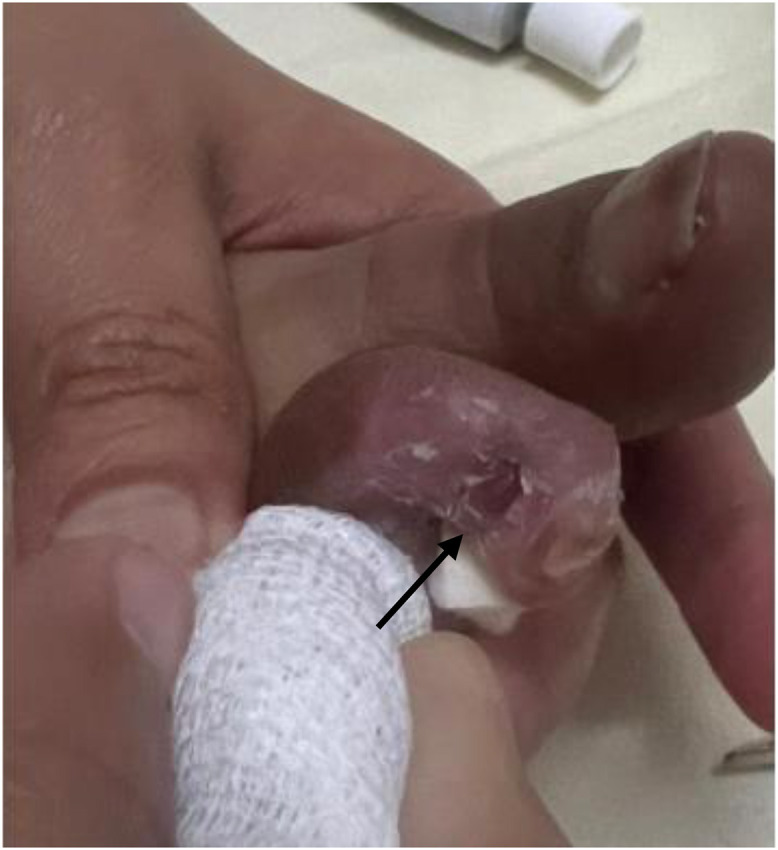
Right foot one year after thyroidectomy, showing dusky discoloration of the right second toe, with a 0.4×0.3 cm open wound (black arrow) on the lateral aspect and granulation tissue on the lateral aspect.

**Table 1.** Laboratory findings on initial presentation.

Laboratory values with units	Patient	Reference
WBC count (×10^3^/uL)	5.0	(4.0–10.0)
RBC count (×10^6^/uL)	4.9	(3.8–4.8)
Hemoglobin (g/dL)	13.4	(12.0–15.0)
Platelets (×10^3^/uL)	294	(150–400)
ESR (mm/h)	36	(2–37)
CRP (mg/L)	<2.0	(0–5.0)
Urea (mmol/L)	4.5	(2.5–7.8
Creatinine (umol/L)	60	(44–80)
ALP (U/L)	86	(35–104)
AST (U/L)	29	(0–32)
ALT (U/L)	17	(0–33)
ANA (titer)	Positive (1:1,280)	1:40
Anti-dsDNA antibodies (IU/mL)	Positive (19)	<16
Anti-Smith antibodies	Negative	
Rheumatoid factor	Negative	
Anti-cyclic citrullinated peptide antibodies	<8	
Complement C3 (g/L)	1.22	(0.90–1.80)
Complement C4 (g/L)	0.14	(0.10–0.40)
Anti-Ro antibodies	Negative	
Anti-La antibodies	Negative	
Anti-centromere antibodies (IU/mL)	Positive (>240)	<7
Anti-Ribonucleoprotein (RNP) antibodies	Negative	
Anti-Jo-1 antibodies	Negative	
Anti-ribosomal P antibodies	Negative	
Anti-neutrophilic cytoplasmic antibodies	Negative	
Lupus anticoagulant	Negative	
Anti-B2 glycoprotein antibodies	Negative	
Anti-cardiolipin antibodies	Negative	

WBC, White blood cells; RBC, Red blood cells; ESR, Erythrocyte sedimentation rate; CRP, C-Reactive protein; ALP, Alkaline phosphatase; AST, Aspartate aminotransferase; ALT, Alanine aminotransferase; ANA, Anti-nuclear antibodies.
